# Tribological and Dynamical Mechanical Behavior of Prototyped PLA-Based Polymers

**DOI:** 10.3390/ma13163615

**Published:** 2020-08-15

**Authors:** Dumitru Nedelcu, Simona-Nicoleta Mazurchevici, Ramona-Iuliana Popa, Nicoleta-Monica Lohan, Demófilo Maldonado-Cortés, Constantin Carausu

**Affiliations:** 1 Department of Machine Manufacturing Technology, “Gheorghe Asachi” Technical University of Iasi, Str. Prof. Dr. Doc. Dumitru Mangeron, No. 59A, 700050 Iasi, Romania; simona0nikoleta@gmail.com (S.-N.M.); poparamonaiuliana1983@gmail.com (R.-I.P.); constantin.carausu@academic.tuiasi.ro (C.C.); 2 Academy of Romanian Scientists, Str. Ilfov, No. 3, Sector 5, 060274 Bucharest, Romania; 3 Department of Materials Engineering and Industrial Safety, “Gheorghe Asachi” Technical University of Iasi, Str. Prof. Dr. Doc. Dumitru Mangeron, No. 71A, 700050 Iasi, Romania; nicoleta-monica.lohan@academic.tuiasi.ro; 4 Engineering Department, University of Monterrey, 66238 San Pedro Garza Garcia, Monterrey, Mexico; demofilo.maldonado@udem.edu

**Keywords:** 3D printing, biodegradable materials, DMA, DSC, SEM, FTIR, TGA

## Abstract

It is essential to combine current state-of-the-art technologies such as additive manufacturing with current ecological needs. Due to the increasing demand for non-toxic biodegradable materials and products, human society has been searching for new materials. Consequently, it is compulsory to identify the qualities of these materials and their behavior when subjected to various external factors, to find their optimal solutions for application in various fields. This paper refers to the biodegradable Polylactic acid (PLA)-based filament (commercially known as Extrudr BDP (Biodegradable Plastic) Flax) compared with the biodegradable composite material PLA-lignin filament whose constituent’s trade name is Arboblend V2 Nature as a lignin base material and reinforcement with Extrudr BDP Pearl, a PLA based polymer, 3D printed by Fused Deposition Modeling technology. Certain mechanical properties (tensile strength, bending strength and DMA—Dynamic Mechanical Analysis) were also determined. The tribology behavior (friction coefficient and wear), the structure and the chemical composition of the biodegradable materials were investigated by SEM—Scanning Electron Microscopy, EDX—Energy Dispersive X-Ray Analysis, XRD—X-Ray Diffraction Analysis, FTIR—Fourier Transform Infrared Spectrometer and TGA—Thermogravimetric Analysis. The paper also refers to the influence of technological parameters on the 3D printed filaments made of Extrudr BDP Flax and the optimization those of technological parameters. The thermal behavior during the heating of the sample was analyzed by Differential scanning calorimetry (DSC). As a result of the carried-out research, we intend to recommend these biodegradable materials as possible substituents for plastics in as many fields of activity as possible.

## 1. Introduction

The use of biodegradable plastics in an increasingly diverse range of applications has required a thorough investigation of their thermal, mechanical and other behavior, in order to provide a complete functional characterization to offer the most satisfying answers to the industrial and scientific needs and challenges.

The main strength of biodegradable plastics is the considerable diminution of the negative effects on the environment, especially when they are disposed of and become municipal waste. One should note that biodegradable plastics are not intended to be stored directly in nature, it would be desirable that their biological decomposition be carried out in a controlled environment, under aerobic conditions (in the presence of air or oxygen, where carbon is transformed into carbon dioxide and biomass) or anaerobic conditions (in the absence of air or oxygen, where carbon is transformed into methane and biomass) and under the action of living organisms [1].

Rapid prototyping is an additive manufacturing technology that determines raw materials economics but also the possibility of obtaining a prototype in a very short time without the need to involve other traditional processing technologies. The permanent desire to develop and refine technologies, whether it refers to modern manufacturing technologies or technologies to obtain materials with low environmental impact, has led to the creation of globally sustainable products. The most widespread technology for obtaining parts by additive manufacturing is FDM (Fused Deposition Modeling), which offers the possibility to obtain prototypes of various sizes in a very short time, unlike conventional manufacturing technologies.

Arboblend V2 Nature is a lignin base thermoplastic material produced by Tecnaro [2]. The Extrudr BDP Pearl and Extrudr BDP Flax—PLA-based filaments are products manufactured by Extrudr and they have a very high rate of biodegradation, due to the fact that their composition is based on renewable raw materials such as annual plants (hemp, sesame and others) and natural additives needed for processing (natural resins) [3].

The area in which the printed biodegradable material samples can find their applicability is very wide, from applications in the automotive industry (parts that are currently produced from non-biodegradable plastic material, parts that relate to the aesthetic side or parts that enter in a gear structure, and which can be replaced with a biodegradable material with similar properties), to applications in the field of electronics, home appliances, toys, furniture and many others.

## 2. Materials and Method

The materials studied in this research belong to the materials category that diminishes the negative global impact and effect of conventional plastics. The selected materials will degrade by fragmentation during the decomposition cycle, after which the products of this decomposition are mineralized by microorganisms and re-enter the natural cycle. Thus, Arboblend V2 Nature and the Extrudr BDP (Biodegradable Plastic) Pearl and Extrudr BDP Flax group are thermoplastics with a very high rate of biodegradability, being made of various renewable raw materials depending on the characteristics desired by their manufacturer.

The group of materials produced by the Extrudr company (Extrudr BDP Flax and Extrudr BDP Pearl) is made from renewable resources, thus being biodegradable (according DIN EN ISO 14855), consisting of the PLA (polylactic acid)–basic matrix, copolyester and additives. The raw material is approved according to the REACH—Registration, Evaluation, Authorisation and Restriction of Chemicals (a European Union regulation), RoHS—Restriction of Hazardous Substances Directive and FDA—Food and Drug Administration Standards. The other constituent elements indicated by the manufacturer are copolyester and additives [3]. Additionally, the Extrudr BDP Flax material contains mineral fillers that lead to a significant increase in the production speed as well as the extrusion temperature. The materials were specially created to be prototyped and used in order to obtain sophisticated design products and not only. The ARBOBLEND^®^ (Arboblend V2 Nature) material is a lignin based product but it has in its composition depending on the formula, biopolymers such as: polylactic acid (PLA); polycaprolactone (PCL); polyester (e.g., bio-PET); polyhydroxyalkanoates (PHA); bio-polyamides (bio-PA); bio-polyolefins (bio-PE); cellulose; starch; natural waxes, resins, fatty acids, oils; natural reinforcing fiber and organic additives, [2].

The paper aims to compare various properties (mechanical, tribological, structural and thermal) obtained following a study of the PLA-based filament (hereinafter referred to it according to the manufacturer as the Extrudr BDP Flax material) and of the composite material PLA-lignin filament, named in the manuscript as Arboblend V2 Nature reinforced with Extrudr BDP Pearl (80% with 20% in weight), in order to establish some recommendations regarding their use as substitutes for non-biodegradable plastics. It should be mentioned that the tested samples were obtained by extrusion (filament obtaining) and 3D printing (Fused Deposition Modeling technology) in the reinforced material case and only by 3D printing in the Extrudr BDP Flax material case.

During the filament extrusion process of the reinforced material, the following adjustable parameters were used on the equipment Noztek Touch and Touch HT (produced by Noztek, Shoreham-by-Sea, UK): T1 temperature of the first resistor sleeve type 150 °C; T2 temperature of the second sleeve type resistor 155 °C; screw rotation speed 8–30 rpm and throughput 2.5 m/min.

By choosing T1 temperature, the extrusion temperature of the material is determined before being pushed through the nozzle hole. A temperature that is too low decreases the material fluidity and the flow rate of the filament. Additionally, its diameter can increase, exceeding the acceptable value. Higher T1 temperature drops may block the material flow through the nozzle. Thus, filament production is halted.

A T1 temperature that is too high results in a thin filament with or without variations in diameter. Due to overheating, the filament continues to flow out of the nozzle.

The T2 temperature allows material granules transformation into a fluid state, fluid material transportation through the cylinder by turning the worm screw and fluid composition homogenization. The T2 temperature must ensure the fluid state of all granules (if they are made of different materials) so as to homogenize the mixture by rotating it and to remove the air initially found between granules.

The rotation speed of the worm screw allows the material to be carried to the extrusion area, its homogenization in a fluid state and the achievement of the pressure necessary to pass the material through the nozzle. The output speed (production speed) of the extruded filament is set by this pressure. Rotation speed increase leads to the increase of frictional forces between material and cylinder and between material and screw. It also leads to the acceleration of the wear of the extruder components, increased forces and extrusion pressure, and consequently to motor overstress.

The process parameters taken into consideration during the prototyping process of the Extrudr BDP Flax parts were: printing temperature 190 °C, heated bed 55 °C, nozzle diameter 0.4 mm and deposited layer thickness 0.15 mm. Additionally, other process parameters considered were the infilling degree 100%, the type of filling in the raster area was grid and in the shell area was rectilinear. The used equipment was Raise3D Pro2 Plus (RAISE3D, Irvine, CA, USA).

The equipment and parameters used during material analysis were as follows:

1. Instron 3382, according to ISO 527–3:2003 recommendation, with a 5 mm/min constant crosshead speed, was used as universal testing equipment to perform the sample tensile test;
2. For determining the bending resistance—WDW 50E universal testing machine (MC, Tianjin, China), using a 1 kN load and 2 mm/min loading rate, tested sample dimensions 50 mm × 4 mm × 10 mm;
3. The dynamic mechanical analysis was performed with DMA 242 Artemis NETZSCH equipment (NETZSCH, Selb, Germany), using the three-point bending method. The temperature scans were performed from room temperature to 373.15 K every three Kelvins, with a dynamic force of 5 N, a deformation of 50 μm and a frequency of 1 Hz;
4. The wear and Friction Coefficient (COF) were measured using a T-11 Elevated Temperature Pin-On-Disk Testing Machine (Tribologia, Radom, Poland) for tribotesting of lubricants and engineering materials, according to the ASTM G99-04 standard test method for wear testing with a pin-on-disk apparatus. The software used to interpret the acquired data was Alicona Edgemaster (Alicona Imaging GmbH, Graz, Austria) from the University of Monterrey, Engineering Department. The following conditions were set at the beginning of the tribological tests: analysis performed under dry slip conditions, ball fixed to a holder and the holder made a circular movement over the square sample, applied load 49 N, angular speed 40 rpm, wear track radius 5 mm, testing time 900 s and ball characteristics (roughness 25 µm; hardness 60 HRC; diameter 12.7 mm and material AISI 52,100 steel);
5. The Differential Scanning Calorimetry (DSC) analysis used a NETZSCH calorimeter type DSC 200 F3 Maia (NETZSCH, Selb, Germany), printed sample fragments smaller than 5 mm and weighing less than 50 mg. The equipment was calibrated using the Bi (Bismuth), Sn (Tin), In (Indium) and Zn (Zinc) standards. The scans were performed within the 20–200 °C temperature range, at the 10 K/min heating rate, and under the Argon protective atmosphere. The used software was Proteus, provided by NETZSCH (version 4, Selb, Germany), and the tangent method was employed. When this method was used, the transformation onset temperature—T_s_ and the transformation end temperature—T_f_ were determined, these values corresponding to the theoretical transformation temperatures. T_50_ marks the point where half of the transformation took place (50%). A linear baseline using the same program was used to determine the amount of specific dissipated/absorbed heat;
6. The surface (SEM), structure (XRD) and chemistry (EDX) analyses were determined using a QUANTA 200 3D SEM-FIB electron microscope (FEI Company, Fremont, CA, USA) and an X-ray Diffractometer X’Pert PRO MRD (Panalytical, Almelo, Holland) for X-ray analysis of the samples;

For the Thermogravimetric Analysis a thermobalance STA 449F1 Jupiter (Netzsch, Selb, Germany) was used. The thermal analysis was performed using a thermogravimetric balance model STA 449F1 Jupiter (Netzsch, Selb, Germany). The thermogravimetric balance was calibrated on temperature and sensitivity with standard metals (In, Sn, Bi, Zn and Al) from 25 to 700 °C. The samples mass was in the range of 7–10 mg and were heated with heating rate of 10 °C/min. The nitrogen (99.999% purity) as carrier gas with a flow rate of 50 mL/min and protective purge for thermobalance of 20 mL/min was used. The samples were heated in open Al_2_O_3_ crucible and Al_2_O_3_ as reference material was used. Data collection was carried out with Proteus^®^ software (NETZSCH, version 5, Selb, Germany).

FTIR-ATR (Fourier transform infrared spectrometer—attenuated total reflection) spectrum was recorded using a spectrophotometer Vertex 70 model (Bruker, Rheinstetten, Germany) in the range of 4000–400 cm^−1^ with 4 cm^−1^ resolution and scan rate 32. The spectrophotometer is equipped with MIRacleTM ATR (PIKE, Madison, WI, USA) accessory designed for single or multi-reflection attenuated total reflectance. The ATR crystal plate is from Diamond (1.8 mm diameter) and solid materials can be put into intimate physical contact with the sampling area through high-pressure clamping, yielding high-quality reproducible spectra.

The influence of technological parameters was determined using the MiniTab software (17.1.0 version, Minitab, LLC, State College, PA, USA), the ANOVA factorial method, and the TableCurve3D v.4.0 (SYSTAT Software Inc., San Jose, CA, USA) application was used for the optimization of the technological parameters in the 3D printing with filament from biodegradable materials.

## 3. Results and Discussion

### 3.1. Mechanical Properties

#### 3.1.1. Tensile Tests

The Extrudr BDP Flax samples were 3D printed at an extruder temperature of 190 °C and at a printing table temperature of 55 °C. Table 1 shows the experimental results of the tensile test for the Extrudr BDP Flax material.

**Figure 1:**
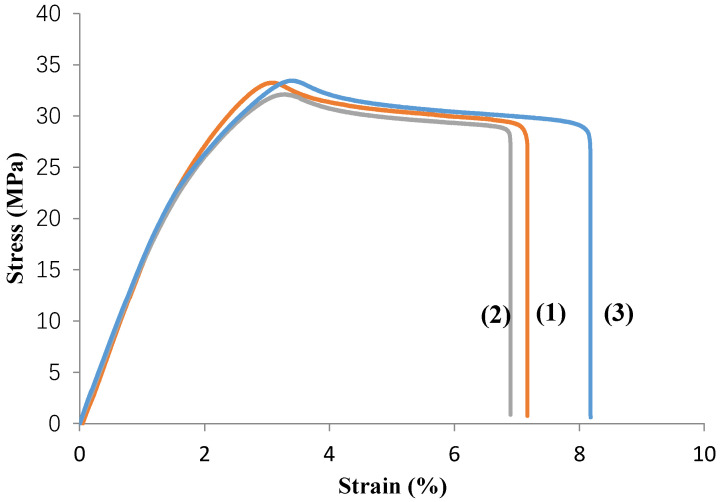
Stress-strain curves, for the Extrudr BDP Flax material, experiment no. 6.

The specific curves similitude of each experiment revealed (example exp. no. 6, Extrudr BDP Flax) the relatively homogeneous behavior of the samples (curves 1–3), Figure 1, which shows that the printing process was stable and reproducible, the mechanical properties of the material varying within acceptable limits. For example, in the experiment with the best results, experiment number 6, the value of tensile strength, σ_M_, reached 32.96 ± 0.72 MPa, the tensile strain at tensile strength, ε_B_, was 3.24% ± 0.14% and the total extension achieved at the final moment of the test, ε_t_, 7.27% ± 0.45%. The obtained results of the prototyped samples are comparable to those of some prototyped parts made of conventional plastics, that is if we do not take into account the process parameters established for each of them, namely being flexible—referred to as TPE (Thermoplastic Elastomers—a blend of plastic and rubber, e.g., Ninjaflex) or TPU (Thermoplastic Polyurethane) with values between 26 and 43 MPa, ABS (Acrylonitrile Butadiene Styrene) 33 MPa [4], HIPS (High Impact Polystyrene) and PP (Polypropylene) 32 MPa [5] and others.

Since no samples of Arboblend V2 Nature composite material reinforced with 20% Extrudr BDP Pearl have been printed and tested on traction so far, no recommendations can be made regarding the replacement of some plastic parts with others made of reinforced biodegradable material, if only this mechanical characteristic is taken into account.

#### 3.1.2. The Influence of Technological Parameters on the 3D Printed Filaments Made of Extrudr BDP Flax

Table 2 shows the results of the ANOVA analysis regarding the factors’ influence on tensile strength. For the studied material, Extrudr BDP Flax, sample orientation (the Fisher value being 168.17) had the largest and statistically significant influence (*p* < 0.001), followed by the deposited layer thickness (*p* = 0.003 and Fisher value = 41.24). The printing speed parameter did not have a statistically significant influence (*p* > 0.05).

**Figure 2:**
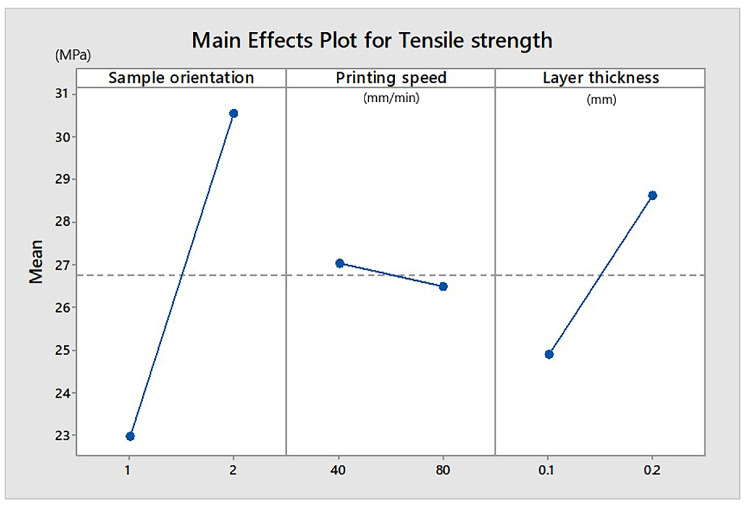
Influence of parameters on tensile strength (Extrudr BDP Flax samples). Sample orientation: 1—plan and 2—on edge.

Figure 2 shows the factors’ main effects on tensile strength. Higher tensile strength values were evidenced by the samples printed on edges and those with thick deposited layers (0.2 mm). Low printing speed (40 mm/min) would provide greater tensile strength.

Table 3 shows the results of the ANOVA analysis regarding the factors’ influence on the modulus of elasticity. Again, the most important and statistically significant influence (*p* = 0.001) is sample orientation (Fisher value being 97.54). The other two factors (layer thickness and printing speed) had no statistically significant influence (*p* > 0.05).

**Figure 3:**
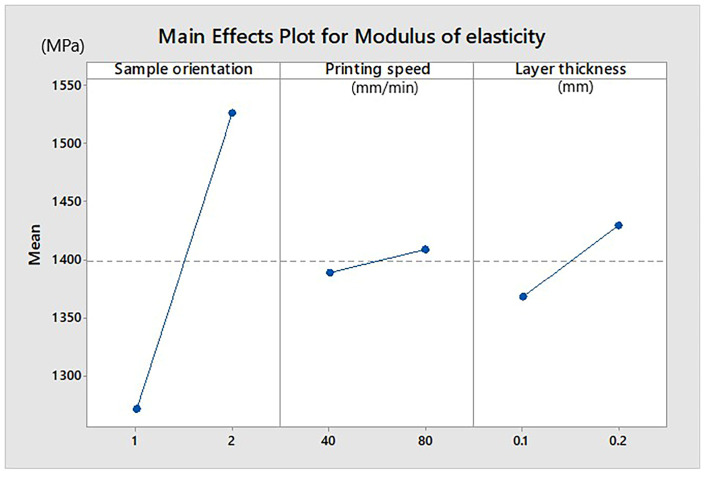
Influence of parameters on modulus of elasticity (Extrudr BDP Flax samples). Sample orientation: 1—plan and 2—on edge.

The effects of the main factors on the modulus of elasticity, E, are shown in Figure 3. The higher modulus of elasticity had the samples placed on edge. A larger thickness of the printed layer offers a greater elasticity modulus. The printing speed parameter had no visible influence.

The results of the ANOVA analysis regarding the process parameters’ influence on elongation, ε are shown in Table 4. Neither factor has a statistically significant influence, having a *p*-value above 0.05.

**Figure 4:**
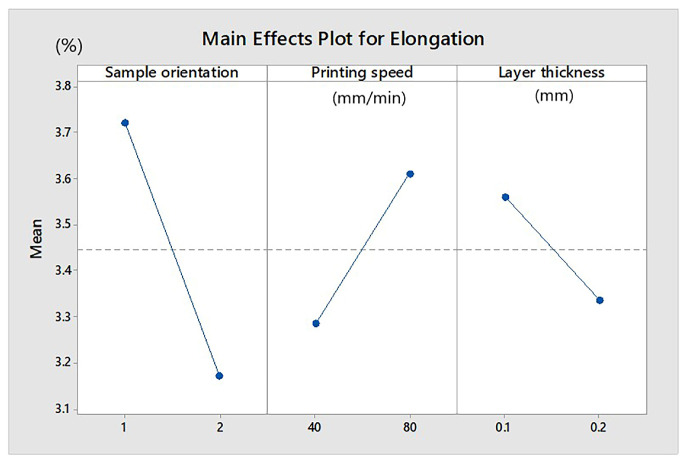
Influence of parameters on elongation (Extrudr BDP Flax samples). Sample orientation: 1—plan and 2—on edge.

The influence of parameters on elongation for the samples printed from Extrudr BDP Flax material is graphically represented in Figure 4. Higher elongation was specific to samples made at high printing speeds and with thinner printing layers (0.1 mm). Elongation increased for the plan-oriented samples on the printing table.

#### 3.1.3. Optimization of Technological Parameters in the Case of 3D Printing with Extrudr BDP Flax Filament

The optimization criteria considered were the maximization of tensile strength, modulus of elasticity and elongation.

The optimizations of the technological parameters for Extrudr BDP Flax prototyping are schematically shown in Table 5. For optimization, quantitative technological parameters were taken into account: printing speed, s (mm/s), and layer thickness, t (mm). The printing direction was considered on edge, for which, according to the factors’ influence analysis, the best results for tensile strength, modulus of elasticity and elongation were obtained.

**Figure 5:**
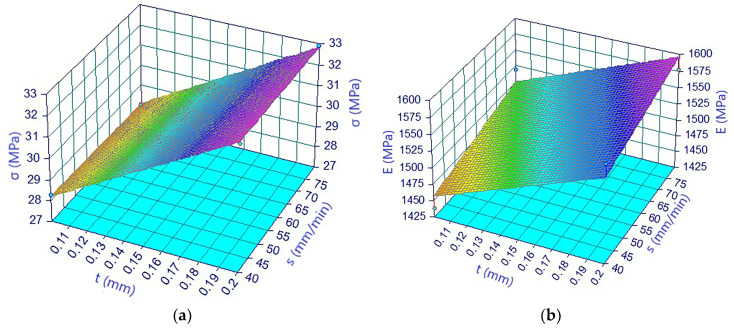

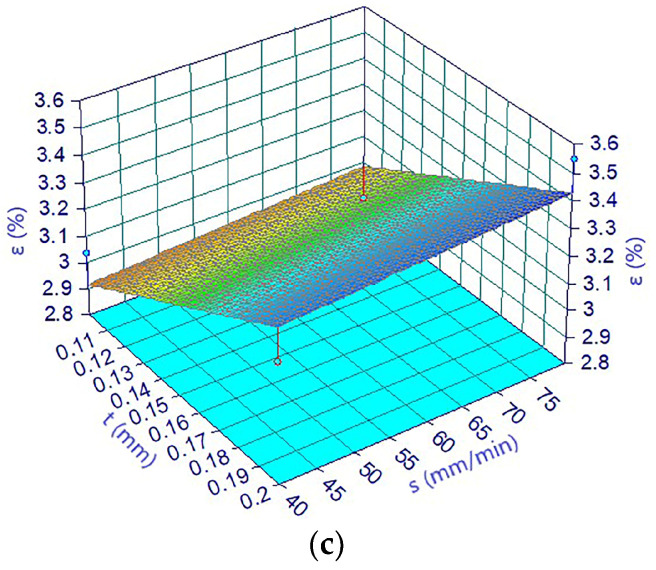
The regression equation for: (**a**) tensile strength; (**b**) modulus of elasticity; (**c**) elongation and samples printed on the edge from the Extrudr BDP Flax filament.

Thus, Figure 5a shows the plan obtained by regression, which underlines the influence of printing speed and layer thickness on tensile strength. The correlation coefficient was very good 0.999, deviations from the experimental points being very small. In Figure 5b,c, graphs are the regression equations plotted plane, for the modulus of elasticity and for elongation respectively. The correlation coefficient was lower, 0.88 for the modulus of elasticity and 0.77 for the elongation, with an increase of deviations from the experimental points of 1.2% in the case of the modulus of elasticity and of more than double, 3.49%, for elongation.

#### 3.1.4. Bending Tests

Following the bending test of a single sample from the studied biodegradable material, an elongation displacement higher than 10% was recorded, which makes the material unsuitable for this type of mechanical test because it does not break easily under the action of progressive loading, as it is a ductile material. The maximum bending strength for the tested sample was about 43 MPa and the elongation resistance about 15.4%.

Regarding the bending test of the Arboblend V2 Nature material, results have not been obtained so far and no recommendation can be provided. The behavior of the printed samples of the material used as a reinforcement, Extrudr BDP Pearl, was similar to the Extrudr BDP Flax discussed in this paper, with values of elongation of more than 20%.

#### 3.1.5. Mechanical Analysis in the Dynamic Regime (DMA Test)

**Figure 6:**
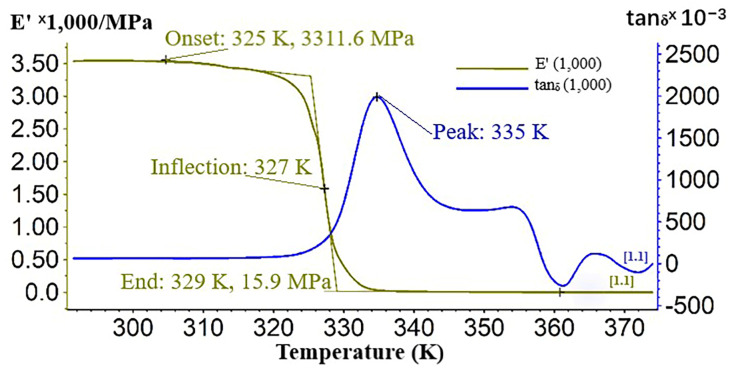
Dynamic Mechanical Analysis (DMA) thermogram recorded during heating of Extrudr BDP Flax.

The DMA diagrams, recorded during temperature scans, display the variations of storage modulus (E′) and internal friction (determined as the ratio between loss and storage modulus, tan d = E″/E′), during a heating cycle. Figure 6 illustrates, through the DMA thermogram, the variations of storage modulus and internal friction during heating.

The values of the Dynamic Mechanical Analysis (DMA) of Extrudr BDP Flax biodegradable material are shown in Table 6, where: E′—storage modulus (MPa), tan_δ_ —damping (×10^−3^), E″—loss modulus (MPa) and T—testing temperature.

The elastic response of the Extrudr BDP Flax material was 3312 MPa, which was recorded at 325 K. Damping occurred within the 310–345 K temperature range, with a peak at 335 K, which also corresponded to the glass transition temperature of the material. The loss modulus (E”) had a value of 6739 MPa.

Analyzing the curves shape of the storage modulus and of the damping that characterizes the Extrudr BDP Flax material, we noted that the material had a sharp and narrow peak for the tan_δ_ curve, which means that the material was homogeneous and had behaved as a unitary whole. The shape of damping peaks was also closely related to the crosslink density of the material, which was confirmed by a thermal and structural analysis. If crosslink density is high, then the material is homogeneous and the damping peak is sharp and conversely it appears as broad peaks. This conclusion is also confirmed by the E’ curve shape, which decreases sharply, almost vertically, which means that phase change occurred within a very short time and temperature. However, the small peaks that appear on the internal friction curve should not be neglected, they denote the fact that there were several phases that were transformed in turn, probable due to water evaporation during polymer heating, decomposition of dyes or additives that enter in the material’s chemical composition.

From the mechanical analysis in the thermal regime point of view, the Extrudr BDP Flax material can replace the ABS (acrylonitrile butadiene styrene) material since its damping value was 2.2 [6].

Comparing the two printed filaments Extrudr BDP Flax and Extrudr BDP Pearl from the point of view of the mechanical behavior obtained during dynamic testing, we concluded that Extrudr BDP Flax was more viscous, due to the fact that its internal friction peak was higher (tan_δ_ = 2.035) than that of the ranfort material, the latter being more elastic (tan_δ_ = 0.478). We specified that the test conditions were the same as for the Extrudr BDP Flax material, including the test temperature (room temperature, 23 °C).

#### 3.1.6. Friction Coefficient

For the COF and wear determination of the studied biodegradable materials, three samples of each type of material were tested in order to confirm experiment stability.

The tribological tests were carried out under dry slip conditions, the tested samples were 20mm × 20 mm in size, and a steel (AISI 52100) ball fixed on a holder performed circular movements over the sample area. Other parameters set during the tests were 49 N applied load, 40 rpm angular speed, 5 mm wear track radius and 900 s testing time. As concerns the ball characteristics, its diameter was 12.7 mm, its roughness 25 µm and its hardness 60 HRC.

**Figure 7:**
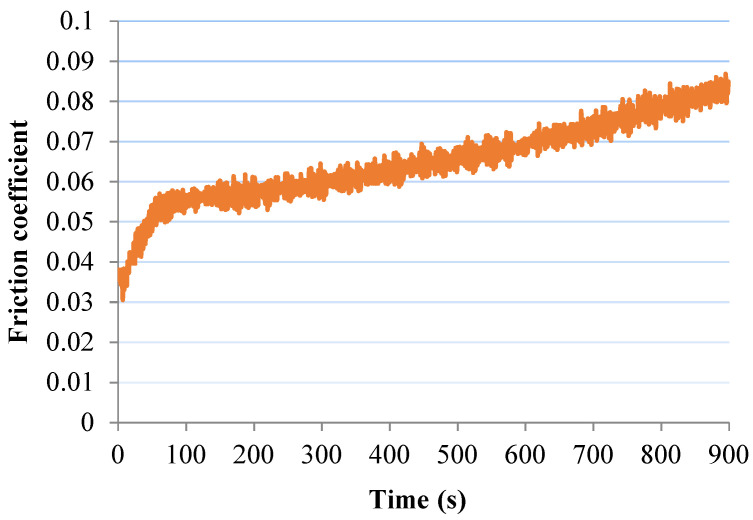
Friction Coefficient (COF) variation with testing time for the Extrudr BDP Flax sample.

According to the graph of the friction coefficient variation with time, Figure 7, obtained by applying a third-degree polynomial equation to the recorded results, the COF value of the Extrudr BDP Flax material increased in the first 60 s from the static value of 0.03 to the value of 0.58. Then, its value gradually increased to 0.86 in 881 s.

COF determination of the printed Extrudr BDP Pearl material was not possible as the sample was destroyed due to the very high friction between the steel ball and the sample surface.

#### 3.1.7. Wear

**Figure 8:**
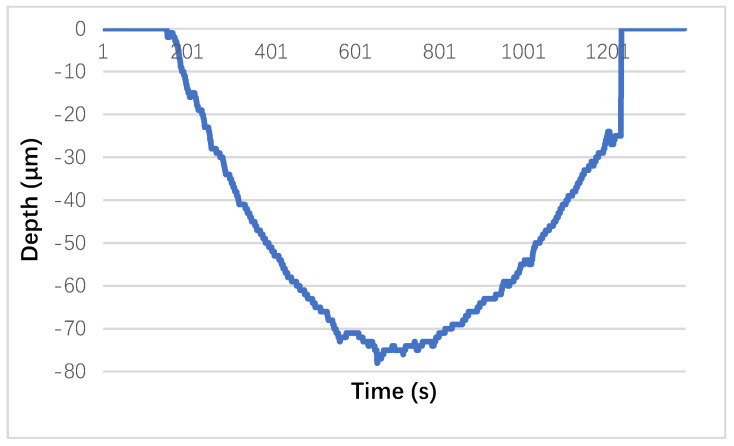
Average wear variation for the Extrudr BDP Flax material.

Figure 8 shows the trace left by the steel ball at the time of cyclic displacement on the Extrudr BDP Flax sample surface. According to the graph, the average wear value resulting from tribological testing was 53.2 ± 5.2 µm, the values recorded for each of the three tested samples being mentioned in Table 7.

**Figure 9:**
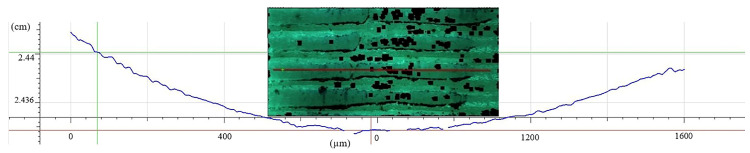
Image of a section of the worn area and variation recorded when wear occurred on the Extrudr BDP Flax material.

Additionally, the worn area, plastically deformed during the testing, is shown in Figure 9 together with the depth variation left by the steel ball on one of the sample’s surfaces. The maximum depth reached during surface use was −74 µm.

The wear behavior of Arboblend V2 reinforced with Extrudr BDP Pearl could not be determined, for the same reason as in the case of COF determination, namely the destruction of the sample before this characteristic could be established.

### 3.2. Morphology and Structure

#### 3.2.1. SEM Analysis

**Figure 10:**
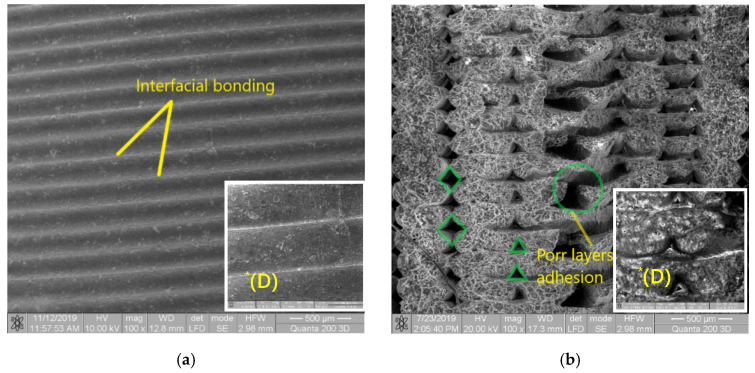
SEM images of the sample prototyped from Extrudr BDP Flax. (**a**) SEM image on the sample surface. (**b**) SEM image in the breaking area. *(D) detail, 500×.

The surface image of the Extrudr BDP Flax sample, Figure 10a, shows a homogeneous surface with filaments deposited in a specific mode of successive printing at a 45° and −45° angle. At the same time, the bonding and adhesion between the extruded and deposited filaments may also be noted in the detailed image.

Comparing within the cross-section of the sample prototyped from Extrudr BDP Flax, Figure 10b, the size of the triangular holes in the raster area and the size of the rhomboid holes in the edge of the specimen (where individual layers of filament form the so-called shell layer), noted, contrary to our expectations, that in the raster area the specimen has higher porosity and sometimes the layers lack adhesion. This inner structure indicates a rapid cooling of the material, which prevented the extruded filament from filling the free space of the model. In order to reduce model porosity, the authors of reference [7] recommend a significant increase (even double) of the printing table temperature and, if possible, of the print nozzle temperature.

For the reinforced material, SEM images were recorded only to determine the structure of the filament obtained by extrusion; its structure is homogeneous, without voids or non-embedded areas, unmolten Extrudr BDP Pearl granules in the Arboblend V2 Nature material.

#### 3.2.2. EDX Chemical Analysis

**Figure 11:**
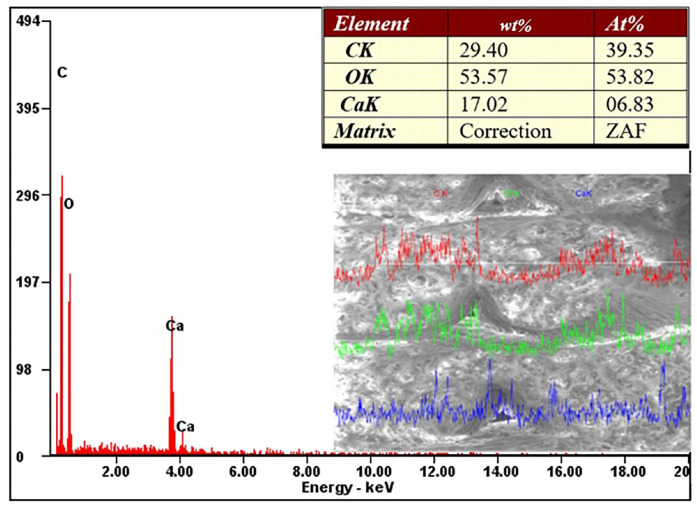
EDX spectroscopic analysis for the Extrudr BDP Flax material.

Figure 11 shows the mass and atomic percentage of the chemical elements in the Extrudr BDP Flax prototyped sample. The main chemical elements identified both in the EDX graph and in the in-line analysis of a sample area were carbon, oxygen, and calcium. The mass percentages were approximately 54% oxygen, 29% carbon and 17% calcium.

The chemical characterization realized by the EDX analysis revealed elements that are found in abundance in the chemical composition of annual plants (raw material—vegetable plants), elements that are biodegradable.

#### 3.2.3. Fourier Transform Infrared Spectrometer (FTIR) Analysis

**Figure 12:**
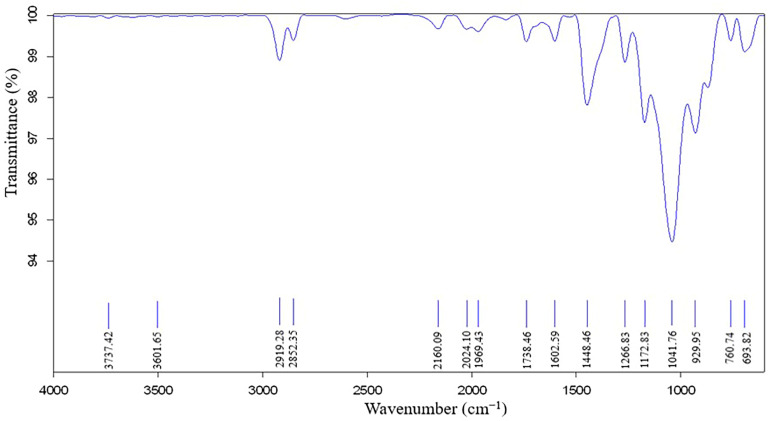
FTIR spectrum of the Extrudr BDP Flax material.

A Fourier Transform Infrared Spectrometer was used in order to provide a molecular fingerprint of the Extrudr BDP Flax material, Figure 12. In the FTIR spectrum of Extrudr BDP Flax, can be observed the presence of transmittances bands characteristic to hydroxyacids, the positions of these bands being confirmed by the literature results [8,9,10]. The PLA base matrix of the material was visible through a band at 1738.46 cm^−1^ stretching of the carbonyl group, the bending vibration appeared at 1266.83 cm^−1^. The amorphous and crystalline phases of polylactic acid corresponded to 760.74 cm^−1^ [11,12].

The peaks registered at 1448.46 cm^−1^ and at 693.82 cm^−1^ are characteristic to the calcium carbonate compound [13,14], highlighted also by the EDX and XRD analyses. Other transmittance bands presented by the spectrum were at 1172.83 cm^−1^—stretching vibrations from COOR groups—and at 1041.76 cm^−1^—deformation vibrations (C–OH) specific to secondary alcohols [10]. The 2919.28 cm^−1^ and 2852.35 cm^−1^ peaks are mainly C-H stretching vibrations of ethylene [15,16] and at 3501.65 cm^−1^ is recorded the transmittance band attributed to the OH groups, [10].

The copolyester incorporated in the base matrix of the Extrudr BDP Flax material as a compatibilizer agent has a chemical structure similar to that of PLA [17], so the transmittances bands largely coincide making it difficult the identification of its specific bands. The similar structure of the compatibilizer agents (copolyester and additives) ensures the chemical compatibility of the compounds, thus making possible the forming of a new material that has a homogeneous chemical structure.

The obtained spectrum confirms that the PLA base matrix of the material due to the fact that this one is similar to that of pure PLA [11] thus confirming the high rate of biodegradability.

Spectral characteristics of Arboblend V2 Nature reinforced with the BDP Pearl Extruder composite material has a high percentage of polyhydroxyalkanoates or other added polyesters/polyethers, the notable exceptions being the presence of OH functions and lignin derivatives. The identified substances are part of the wood, natural plants and natural fibers compounds (lignin, cellulose and hemicellulose) but also of the PLA spectrum. This FTIR analysis confirmed the biodegradable composition of the composite material.

#### 3.2.4. XRD Analysis

**Figure 13:**
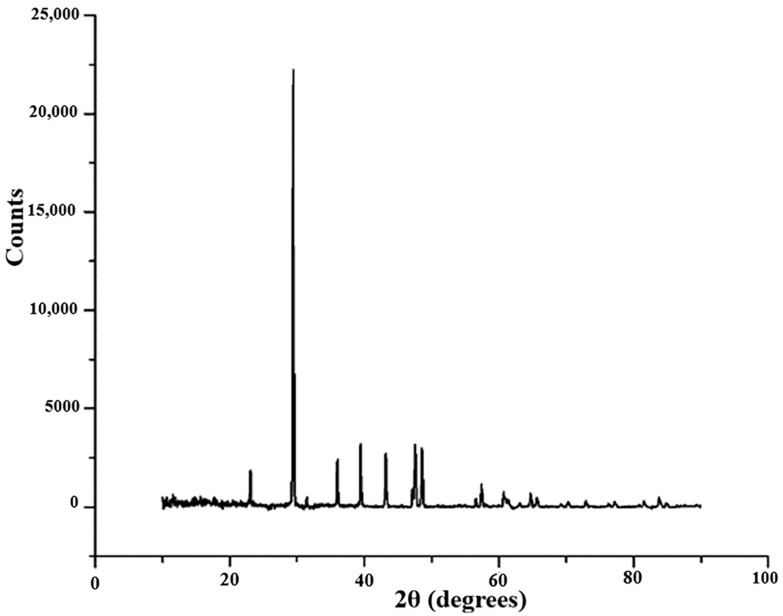
XRD analyses for the Extrudr BDP Flax material.

Figure 13 shows the results obtained by an Energy Dispersive X-ray Analysis (EDX), which helps to identify the elemental composition of an Extrudr BDP Flax printed sample area.

Following the X-ray analysis of the Extrudr BDP Flax sample, Figure 13, a majority peak was identified at 29.43 2θ angles, with an intensity of 22,230, which, according to references [18,19], would be attributed to the presence of calcium carbonate in the material.

The presence of the calcium carbonate compound in the Extrudr BDP Flax material’s chemical composition could most probably be due to the manufacturer’s use of this compound in order to increase the mechanical performance of the material and to decrease its price. It is known that this mineral reinforcement is one of the most used additives in the plastics industry. Its role could be to reduce surface energy and provide opacity and surface gloss, which improves the surface finish. Moreover, when particle size is carefully controlled, CaCO_3_ helps to increase both impact resistance and rigidity—a requirement that becomes important at high usage temperatures. For example, calcium carbonate is used in the production of PVC (polyvinyl chloride), polypropylene (PP), ABS (acrylonitrile butadiene styrene) and other, in order to improve mechanical, electrical and implicitly thermal characteristics such as tensile and elongation resistance, volume resistivity, etc. [20].

Additionally, a smaller peak was revealed at an angle of 22.5° 2θ, Figure 12—first peak, which, according to similar results obtained by the authors [21,22], corresponds to the cellulose crystallographic plane ((002) Miller index).

The identified peaks clearly prove the existence of the crystalline phase in the examined biodegradable thermoplastic material. The identification of all peaks has not yet been possible, however, based on the thermal analysis and the data obtained from the literature, the statement above regarding the occurrence of the crystalline phase was confirmed.

For the Arboblend V2 Nature reinforced with the Extrudr BDP Pearl sample, Figure 13, a majority peak was identified at 16.7 2θ angles, which, according to the authors of the paper [23], could be linked with the presence of lignin (the basic matrix) in the material composition or of vegetable natural fibers, thus confirming that the material had in its composition renewable raw materials and of course that it was biodegradable.

Diffraction analysis revealed the CCaO_3_ base compound, with rhombohedral crystal structure; the lattice parameters of the identified compound are shown in Table 8.

### 3.3. Thermal Analysis

#### 3.3.1. Differential Scanning Calorimetry (DSC) Analysis

In order to determine the transitions that occur in the structure of the biodegradable Extrudr BDP Flax material during heating, thermal analysis was performed by differential scanning calorimetry (DSC). The samples, in the form of the filament, were heated by 10 K/min up to a temperature of 200 °C. The determinations were stopped before the sample underwent unwanted structural changes, such as carbonization, since one of the objectives of the analysis was to determine the temperature range at which the material begins to melt, i.e., the melting point, in order to determine the optimal prototyping temperature.

**Figure 14:**
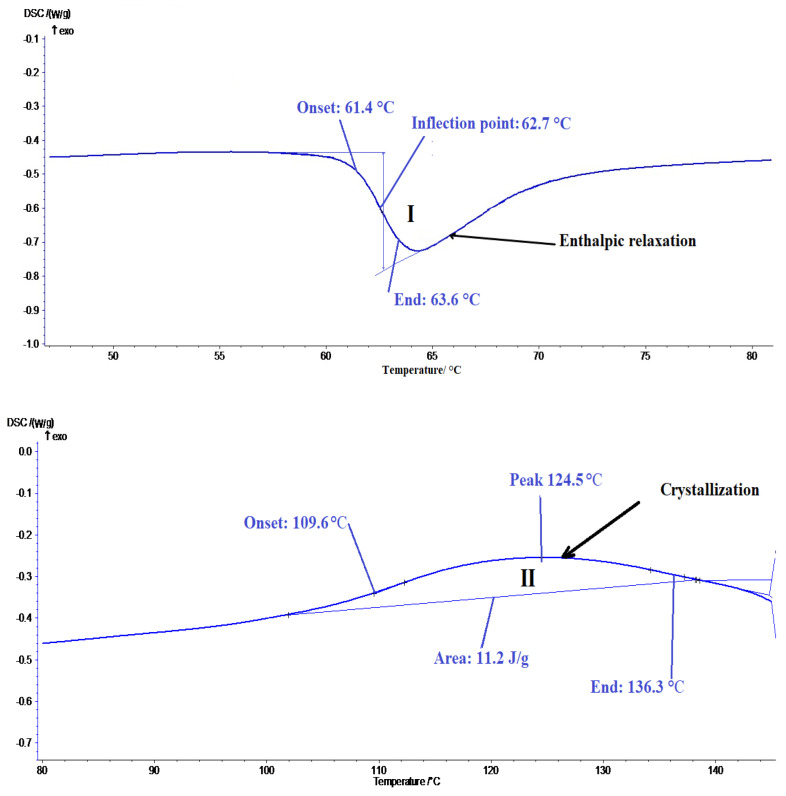

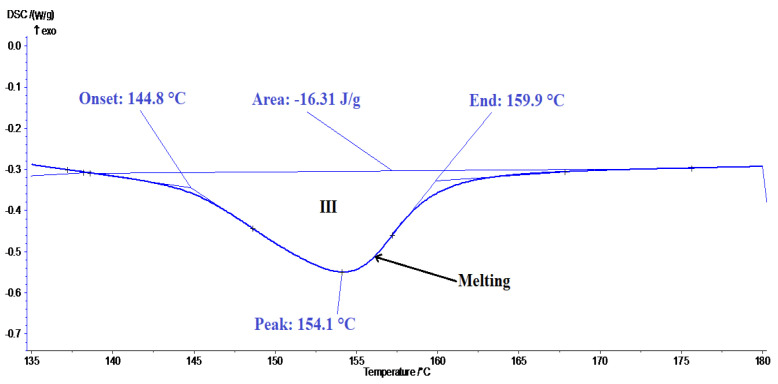
Highlighting the main thermal behavior of the printed Extrudr BDP Flax samples.

Figure 14 shows the DSC thermogram recorded during controlled heating within the 40–190 °C temperature range of the filament made of the Extrudr BDP Flax. Three transformations were revealed by the thermogram during heating: an endothermic slop, one exothermic, accompanied by dissipation heat and one endothermic, accompanied by heat absorption. Following the evaluation, the transformation temperatures and the amount of heat dissipated or absorbed were centralized in Table 9.

For the glass transition critical temperatures (T_onset_—starting temperature, T_mid_—middle temperature, T_inf_—inflection point and T_end_—finish temperature) and heat capacity (ΔCp) was determinated. The critical temperatures of second and third transformations (T_s_—starting temperature, T_50_—middle temperature and T_f_—finish temperature) were determinated using the tangent method and the absorbed/dissipated heat (ΔH) using a linear baseline.

Calorimetric analysis revealed that the Extrudr BDP Flax sample exhibited three representative transformations, the first at 62.7 °C (inflection point), which can be associated with the glass transition of amorphous material components (probably PLA or copolyester), [24,25], followed by the ascending slope, which suggests an enthalpy relaxation, [26,27]. The exothermic peak at 124.5 °C (curve II) that takes place with a heat release within the 109.9–136.3 °C temperature range, could correspond to the hot crystallization of the material, the process taking place with minimal energy consumption [24,25]. The Curve III endothermic peak at 154.1 °C corresponded to the melting point of the material. The temperature range considered in order to determine the optimum 3D printing temperature was 145–165 °C, [26,28].

Regarding the thermal behavior of the reinforced material, it showed, at a temperature of 62.2 °C, occurs a second-order transition, associated to glass transition, temperature at which the material becomes more rigid. The melting temperature of the reinforced material is represented by two endothermal peaks each corresponding to the melting point of the Arboblend V2 Nature material (172 °C) and to the melting point of the reinforcement (Extrudr BDP Pearl) material at 149.4 °C, respectively, [29].

Thus, according to the diffractograms and thermograms of the Extrudr BDP Flax, Arboblend V2 Nature and Extrudr BDP Pearl (results of the authors not published so far) materials, they have a semicrystalline structure [30].

#### 3.3.2. The Thermogravimetric Analysis (TGA)

**Figure 15:**
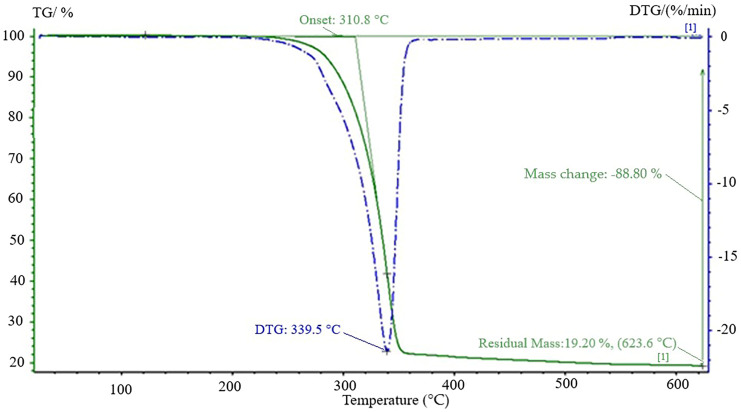
TGA curve of the Extrudr BDP Flax material.

The thermogravimetric analysis of the Extrudr BDP Flax material was performed to highlight its thermal stability of this one, Figure 15. Thus, the thermogram showed a decomposition in a single step where the loosed mass was about 80% at a degradation temperature of 339.5 °C obtained from the DTG curve. The spectrum of the remaining residue after 650 °C was 19.2% and could be associated with the undecomposed solid residue from PLA, but also with the copolyester and additives that were introduced and did not degrade until the end of the analysis. According to the literature, the residual mass left after the thermogravimetric analysis of pure PLA up to temperatures of 650 °C was between 1 and 2%, [31,32]. The difference of about 17% was associated with the non-decomposition of copolyester, which according to the literature takes place in proportion up to 80% at temperatures of about 400 °C, [33], the calcium carbonate mineral additive with decomposition in the temperature range of 600–850 °C [34].

The fact that the material did not lose mass of more than 2% until the melting temperature of the material was reached (154 °C) indicates that it was thermally stable and could be used for prototyping without its structure being affected or influenced even up to temperatures above 200 °C when the chemical bonds begin to break and its structure begins to be damaged.

Comparing the composite material with the Extrudr BDP Flax one from the thermogravimetric point of view had two stages of decomposition, the first at about 330 °C (associated with lignin) [32], which according to the literature can decompose in the 160–900 °C temperature range and the second stage at a temperature of about 400 °C, which can be associated with the decomposition of a constituent of the material, reinforcement–PLA, additives and natural resins—with a decomposition temperature between 450 and 750 °C, [35,36].

## 4. Conclusions

The paper presented the study of the Extrudr BDP Flax material properties compared to the Arboblend V2 Nature reinforced with Extrudr BDP Pearl properties, for samples obtained by 3D printing, using Fused Deposition Modeling technology.

As far as tensile strength is concerned, the results showed that the recorded values are superior to some commonly used plastics, such as thermoplastic polyurethane, acrylonitrile butadiene styrene, high impact polystyrene and polypropylene.

The obtained results confirmed that the process parameters influenced the microstructure of the material. Thus, the deposition of layer thickness led to a rough and ununiformed structure due to the variety of gaps. The layer thickness requires a higher printing speed, which can generate both vibrations at the print head and variations in the pressing force of the deposited layers, which leads to dimensional unevenness of the deposited filament and the density of the microstructure. Going further to the orientation of the sample (parameter with the greatest influence), the shell area and the raster area, influence the physical–mechanical properties due to the geometry of the pattern and the degree of infilling and implicitly the structure.

The melting temperature of the filament and the temperature of the printing bed influenced the sample structure and implicitly its characteristics by using optimal temperatures (which does not lead to mass loss or too fast cooling of the material) that helped to increase the layer’s adhesion and decrease the structure porosity specific to FDM printing.

Thermal analyses performed on the Extrudr BDP Flax material revealed that it was thermally stable around the melting temperature, with no mass loss, which led to the conclusion that printing the material at a temperature of 190 °C did not lead to a rupture between the chemical bonds of the material, which was thermally stable up to a temperature of 250 °C when the mass loss exceeded 2%, and when, according to the literature, the material characteristics began to suffer.

According to the SEM analysis, the structure of the material was homogeneous, presenting gaps specific to 3D printing. The homogeneity of the material being highlighted by the DMA analysis for which the damping peak was high and sharp, and the slope of the storage module was steep. The homogeneity of the material was also confirmed by the FTIR spectrum of the material, which had transmission bands characteristic to base matrix–PLA and the embedded copolymer, which overlapped, indicates that the components incorporation was homogeneous.

The structural analyses EDX, FTIR and XRD certify the presence of a mineral additive, calcium carbonate, incorporated by the manufacturer in order to increase the mechanical and thermal performance of the material but also to decrease its price.

According to the obtained results regarding the characteristics of the printed samples from the Extrudr BDP Flax material, it can be stated that it can replace a series of nonbiodegradable plastics currently used in additive manufacturing such as TPU, ABS, HIPS, PP and so on.

## Figures and Tables

**Table 1:** Experimental results of tensile tests performed on the Extrudr BDP Flax samples.

Experiment Number	Input Parameters			σ_M_ (MPa)	ε_B_ (%)	ε_t_ (%)	E (MPa)
	Layer Thickness (mm)	Printing Speed (mm/s)	Sample Orientation				
1	0.1	40	Plan	22.26 ± 1.37	3.50 ± 0.54	5.38 ± 1.01	1251.47
2	0.1	40	On edge	28.27 ± 0.09	3.04 ± 0.13	3.39 ± 0.48	1437.16
3	0.1	80	Plan	21.07 ± 0.98	4.49 ± 0.50	4.50 ± 0.50	1265.72
4	0.1	80	On edge	27.97 ± 0.89	2.86 ± 0.11	2.98 ± 0.16	1519.13
5	0.2	40	Plan	24.58 ± 0.42	3.36 ± 0.05	7.38 ± 0.51	1293.34
6	0.2	40	On edge	32.96 ± 0.72	3.24 ± 0.14	7.27 ± 0.45	1572.86
7	0.2	80	Plan	24.05 ± 0.49	3.19 ± 0.16	6.83 ± 0.62	1274.90
8	0.2	80	On edge	32.91 ± 0.56	3.55 ± 0.41	7.04 ± 1.16	1577.11

**Table 2:** The variance analysis results by the ANOVA method for tensile strength (Extrudr BDP Flax samples).

Parameter	Freedom Degrees	Square of the Adjusted Amounts	Square of the Adjusted Mean	Fisher Value	Probability of Fisher Value, *p*-Value
Sample orientation	1	113.628	113.628	168.17	<0.001
Printing speed (mm/s)	1	0.536	0.536	0.79	0.424
Layer thickness (mm)	1	27.863	27.863	41.24	0.003
Error	4	2.703	0.676	-	-
Total	7	144.729	-	-	-

**Table 3:** The variance analysis results by the ANOVA method for the modulus of elasticity (Extrudr BDP Flax samples).

Parameter	Freedom Degrees	Square of the Adjusted Amounts	Square of the Adjusted Mean	Fisher Value	Probability of Fisher Value, *p*-Value
Sample orientation	1	130,262	130,262	97.54	0.001
Printing speed (mm/s)	1	841	841	0.63	0.472
Layer thickness (mm)	1	7487	7487	5.61	0.077
Error	4	5342	1335	-	-
Total	7	143,931	-	-	-

**Table 4:** The variance analysis results by the ANOVA method for elongation (Extrudr BDP Flax samples).

Parameter	Freedom Degrees	Square of the Adjusted Amounts	Square of the Adjusted Mean	Fisher Value	Probability of Fisher Value, *p*-Value
Sample orientation	1	0.6050	0.6050	1.46	0.294
Printing speed (mm/min)	1	0.2112	0.2112	0.51	0.515
Layer thickness (mm)	1	0.1013	0.1013	0.24	0.648
Error	4	1.6631	0.4158	-	-
Total	7	2.5806	-	-	-

**Table 5:** Results regarding the technological parameters optimization for the Extrudr BDP Flax printed samples.

Parameter	The Regression Equation	R^2^	Max.	Error/dev. (%)	Optimum	
					t (mm)	s (mm/s)
σ	σ=23.57+48.15·t−0.004·s, (MPa) σ=23.57+48.15·g−0.004·v, (MPa)	0.999	33.04MPa	0.24	0.2	40
E	E=1316.64+968.4·g+1.078·v, (MPa) E=1316.64+968.4·t+1.078·s, (MPa)	0.88	1596.56	1.2	0.2	80
ε	ε=2.408+4.45·t+0.0016·s, (%) ε=2.408+4.45·g+0.0016·v, (%)	0.77	3.426	3.49	0.2	80

where: σ—tensile strength (MPa); E—modulus of elasticity; ε—elongation; R^2^—correlation coefficient; t—layer thickness (mm); s—printing speed (mm/min).

**Table 6:** Results regarding the analysis in the dynamic regime of the Extrudr BDP Flax material.

BDP Flax	E′ (MPa)	tan_δ_ (×10^−3^)	E″ (MPa)	T (°C)
Values	3312	2035	6739	23

**Table 7:** Wear depth values for Extrudr BDP Flax.

Material	Sample	Wear Track Depth (µm)	Average (µm)	Std. Dev. (µm)
Extrudr BDP Flax	1	47.67	53.2	5.2
	2	58.02		
	3	54.05		

**Table 8:** Lattice parameters of CaCO_3._

Compound	Space Group	Crystal System	a (Å)	b (Å)	c (Å)	α (°)	β (°)	γ (°)	Cell Vol (10^6^ pm^3^)
CaCO_3_ Calcium Carbonate (Limestone- calcaros)	R-3c	Rhombohedral	4.9880	4.9880	17.0610	90.0000	90.0000	120.0000	367.61

**Table 9:** Summary of data evaluation with Proteus software.

**Glass Transition**	**T_onset_**	**T_mid_**	**T_inf_**	**T_end_**	**ΔCp**
measurement unit	°C	°C	°C	°C	J/g·K
I	61.4	62.6	62.7	63.6	1.74
**Transformation**	**T_s_**	**T_50_**	**T_f_**		**ΔH/m**
measurement unit	°C	°C	°C		J/g
II	109.6	124.5	136.3		11.2
III	144.8	154.1	159.9		−16.31
